# The Dangers of Cassava (Tapioca) Consumption

**Published:** 1987-05

**Authors:** Michael J Hall

**Affiliations:** University Department of Medicine, Bristol Royal Infirmary, Bristol, BS2 8HW

## Abstract

Cassava (Tapioca) is a worldwide staple food consumed by over 800 million people. It contains cyanide which may lead to acute toxicity or chronically may be an aetiological factor in tropical nutritional amblyopia, tropical neuropathy, endemic goitre, cretinism and tropical diabetes. It may also have carcinogenic potential. However, despite nutritional limitations it has many advantages as a crop to the subsistence farmer and would be difficult to replace.


### The Dangers of Cassava (Tapioca) Consumption

**Michael J Hall, MB, MRCP**
University Department of Medicine, Bristol Royal Infirmary, Bristol, BS2 8HW

### SUMMARY

Cassava (Tapioca) is a worldwide staple food consumed by over 800 million people. It contains cyanide which may lead to acute toxicity or chronically may be an aetiological factor in tropical nutritional amblyopia, tropical neuropathy, endemic goitre, cretinism and tropical diabetes. It may also have carcinogenic potential. However, despite nutritional limitations it has many advantages as a crop to the subsistence farmer and would be difficult to replace.

### INTRODUCTION

Cassava also known as tapioca, manioc or yuca is a staple food for several hundred millions of people worldwide (Hahn and Keyser, 1985). It has been cultivated for between 2500 and 4000 years in the Americas and was discovered by the conquistadores from the Old World (Cock, 1982). Cassava was brought to West Africa from Brazil by the Portugese slave traders of the 16th century (Jones, 1959) its cultivation increasing rapidly in Nigeria and throughout tropical Africa during the 19th and early 20th centuries (Lancaster et al, 1982). It was taken to India in the 17th century and cassava was adopted as a cash crop by the colonial authorities in Southeast Asia in the mid- 19th century, large plantations being established to produce starch and pearl tapioca (Brautlecht, 1953). Indonesia and Thailand are now amongst the largest producers of cassava. In Britain cassava can be found in the markets used by Afro-Caribbeans, such as Brixton in London or St. Pauls in Bristol.

Botanically cassava is known as Manihot esculenta Crantz. There are a large number of cultivars but no botanical distinction between sweet and bitter varieties, so called because of their relative cyanide content, as the two merge into each other with many environmental factors determining the final cyanide composition (Purseglove, 1974).

### THE CROP

Cassava is a short-lived shrub, 1–5 m in height which develops from 3 to 10 tubers per plant for which the crop is predominantly grown. The leaves are also edible, and unlike the roots which are essentially carbohydrate, are a good source of protein and vitamins (Lancaster and Brooks, 1983). Harvesting is carried out between 9 and 24 months depending on the variety (Purseglove, 1974). Cassava will grow between latitudes 30° N and 30° S of the equator, up to 2000 m above sea level, in temperatures from 18° to 20° C and in rainfall of 50 to 5000 mm annually (Okigbo, 1980).

### CASSAVA PRODUCTION WORLDWIDE

World cassava production for 1983 was estimated at 123 million metric tons (Table 1). During the last 20 years, total cassava production has increased at the same rate as population growth in developing countries. This increase is largely due to increases in area planted since yields have remained constant around 9 tons per hectare (far below the maximum experimental yield of 80 tons per hectare (International Centre for Tropical Agriculture, 1979)).

Approximately 65 per cent of the total cassava production is used for direct human consumption and of this, about half is eaten after the fresh roots are cooked and the other half is processed in a number of different ways to make flours or meals. At present approximately 500 million people consume 300 kcals per day as cassava, in Africa 50 million people consume 500 kcal/day and in southern India 25 million people consume over 700 kcal/day (Cock, 1982).

Freshly harvested cassava tubers are more highy perishable than other major root crops but the subsistence farmer's way of overcoming this is to leave the crop in the ground until needed (Richard and Coursey, 1981).

### NUTRITIONAL LIMITATIONS

The nutrient composition is shown in Table 2. The fresh cassava root is mainly water and carbohydrate although it is relatively rich in vitamin C and calcium. It is poor in protein and other vitamins and minerals. The amino acid profile is low in some essential amino acids, particularly the sulphur containing methionine (Okigbo, 1980), and the protein content is reduced still further by the traditional processing methods (Lancaster et al, 1982). Vitamin C content is reduced considerably during processing and in some types of cassava flour no vitamin C remains. Much of the thiamin and niacin found in the raw root is also lost particularly during the washing processes.

Many nutritionists regard cassava, with its almost exclusively carbohydrate content, as unsuitable as a main

**Table 1**

**Cassava production in 1974–76 and 1983**

| | | World | Africa | Asia | Americas |
|---|---|---|---|---|---|
| Area of cultivation | 1974–76 | 12573 | 6745 | 3097 | 2702 |
| ($\times 10^3$ ha) | 1983 | 14879 | 8065 | 4087 | 2705 |
| Yield | 1974–76 | 8564 | 6367 | 10658 | 11640 |
| (kg/ha) | 1983 | 8277 | 5983 | 11237 | 10624 |
| Production | 1974–76 | 107673 | 43002 | 33010 | 31452 |
| ($\times 10^3$ metric tons) | 1983 | 123153 | 48251 | 45929 | 28737 |

Source: FAO Production Yearbook. FAO Rome 1983 vol 37.

staple crop (University of Georgia, 1972) but this is to deny its value as a major source of energy, up to 10% world wide (Anonymous, 1973) and between 25 and 55% in some parts of southern Nigeria (Nicol, 1952).

Clearly in times of drought during which cassava may be the only crop to survive, or other food shortage circumstances, reliance solely on cassava will produce nutrient inbalance and deficiency, but alternatives in such situations do not readily present themselves.

The nutritional contribution of cassava leaves which are a good source of protein and vitamins (Table 2) is frequently ignored in many countries that consume the tubers (Hall, 1986). Unfortunately the leaves are often regarded as poor man's food and only eaten under stress conditions as during the Nigerian Civil War (Anonymous, 1969). Recent analyses have reported protein contents of between 6.3 and 11.8 g/100g fresh weight (Lancaster and Brooks, 1983) which compare favourably with rice and maize. Although the amino acid values of cassava leaf protein exceed those in the FAO reference protein for most essential amino acids, there is a deficiency of the sulphur containing methionine. If the relatively cheap synthetic methionine is added the net protein utilization increases from 32 to 61 per cent (Luyken et al, 1961).

**Table 2**

**Nutritional composition of cassava roots and leaves per 100g**

| | Fresh Cassava root | Leaves (fresh) | Leaves (cooked) |
|---|---|---|---|
| Energy kcals | 149 | 91 | — |
| Moisture % | 62 | 71.7 | 88.3 |
| Protein g | 1.2 | 7.0 | 8.2 |
| Fat g | 0.2 | 1.0 | — |
| Carbohydrate g | 35.7 | 18.3 | — |
| Fibre g | 1.1 | 4.0 | — |
| Ca mg | 68 | 303 | 142 |
| P mg | 42 | 119 | 352 |
| Fe mg | 1.9 | 7.6 | 3.0 |
| Retinol Equivalent μg | — | 2000 | n/a |
| Thiamin μg | 40 | 250 | — |
| Riboflavin μg | 50 | 600 | — |
| Niacin mg | 0.6 | 2.4 | — |
| Ascorbic acid mg | 31 | 311 | 248 |

Source: Food composition table for use in Africa. FAO/US Dept Health. Educ. and Welfare, 1968.

### TOXIC EFFECTS OF CASSAVA CYANOGENETIC GLYCOSIDES

For centuries it has been recognised that cassava may have an acute toxic effect (Clusius, 1605) and that this is related to the content of cyanogenetic glycosides was first demonstrated in 1836 (Henry and Boutron, 1836). Cassava contains 2 cyanogenetic glycosides, linamarin (which accounts for 90% of the total) and lotaustralin. These hydrolyse in the presence of endogenous = linamarinase, released by bruising, soaking etc to liberate hydrogen cyanide (HCN) (Montgomery, 1980). Traditionally cassava is classified as bitter or sweet depending on the cyanogen content, the former having a high level distributed throughout the tuber, but whether there is a close correlation with taste has not been established (Lancaster et al, 1982). Although sweet varieties contain cyanogen it is concentrated mainly in the outer layers, the phelloderm. The cyanide content of the plants is increased in drought (Ministry of Health, Mozambique, 1984), and potassium deficiency (Oke, 1968).

The normal range of cyanogen content of cassava tubers falls between 15 and 400 mg HCN/kg fresh weight (Lancaster et al, 1982) and the traditional methods of processing—drying, boiling, soaking and fermentation—are variably effective at reducing the cyanogen content.

Inorganic cyanide is rapidly absorbed from the gastrointestinal tract and reacts with thiosulphate (for which sulphur containing amino acids are essential (Cliff et al., 1985)) to form thiocyanate. Vitamin B12, as hydroxocobalamin is also essential in the detoxification of cyanide (Osuntokum, 1981).

### ACUTE TOXICITY

The minimum lethal dose of HCN for humans taken orally lies between 0.5 mg and 3.5 mg per kg of body weight (Montgomery, 1980), and deaths after heavy meals of cassava continue to be reported in the Nigerian press (Osuntokun, 1981). Non-fatal acute cases of toxicity have taken the form of drowsiness, weakness and vomiting in children (Cheok, 1978) or irreversible spastic paraparesis (Ministry of Health, Mozambique, 1984). This latter occurrence involved over 1000 persons in Mozambique and was associated with a severe drought which increased the cyanide content of the cassava, the only crop to survive in any quantity. Because of the lack of other foodstuffs there was an absence of sulphur containing amino acids in the diet, and the normal lengthy traditional processing of the cassava was abbreviated.

### CHRONIC TOXICITY

Chronic intoxication is more common in Nigeria and there is now much circumstantial evidence to incriminate the cyanide content of cassava as an aetiological factor in both tropical nutritional amblyopia and tropical neuropathy (Osuntokun, 1981; Ayanru, 1976). In two recent reports, all patients gave a history of longstanding regular cassava consumption, were of low socio-economic status and several had signs of vitamin B deficiencies. None were vitamin B12 deficient (Osuntokun, 1981; Ayanru, 1976).

Endemic goitre and cretinism have been linked to thiocyanate production secondary to cassava consumption. Thiocyanate blocks iodine uptake by the thyroid gland and exacerbates any pre-existing iodine deficiency (Bourdour et al, 1978). Endemic goitre is prevalent in some parts of Nigeria in areas of iodine deficiency and it is likely that cassava consumption is a contributory factor (Kelly and Snedden, 1960).

Tropical diabetes, a disease differing from its Western counterpart in the frequent association with pancreatic calcification, is seen commonly in Nigeria and until recently was felt to be related to poverty and malnutrition. There is now increasing evidence of a link with cassava consumption and ingestion of cyanide (Anonymous, 1979).

Increased thiocynate concentrations in saliva and gastric juice facilitate the production within the stomach of nitrosamines which have carcinogenic potential (Osuntokun, 1981).

### REDUCING CASSAVA TOXICITY

Despite the evolution of cassava processing, many of the traditionally processed foodstuffs still contain appreciable quantities of HCN (Oyefeso, 1976). Some of the newer methods, however, such as screw processing are significantly quicker and more effective (Oben and Menz, 1981). Other strategies to minimise toxicity rely on an awareness of 'at risk' groups. Education during times of drought that full processing procedures should be followed, may be possible. Vitamin B12 deficiency should be guarded against and treated if found, and programmes to improve protein energy malnutrition, with particular attention to sulphur containing amino acids might be instituted. In areas of iodine deficiency, administration of iodised oil has been effective in the prevention of goitre (Cock, 1982).

### ADVANTAGES OF CASSAVA

Despite the dangers and drawbacks both from a nutrient and toxicological standpoint, cassava is one of the most important foodstuffs of the tropical world, and has been described as the 'tropical staff of life' (Rickard and Coursey, 1981). As Hahn and Keyser comment (1985), "How a crop that contributes significantly to the diets of over 800 million people (worldwide) can be virtually unknown beyond its area of consumption is part of the nature of subsistence farming". Cassava is a particularly valuable crop to the subsistence farmer giving a higher energy productivity than other staples (Coursey and Haynes, 1970). It is not season bound, has a low cost of production with low labour requirements and easy cultivation, has the ability to grow in suboptimal soils and acts as a famine security crop available for harvest as needed (University of Georgia, 1972). It is resistant to insect pests particularly the migrating African locust (Wood, 1965).

Cassava also has many advantages as a livestock feed, and numerous non-food uses. There is much scope for increasing the export of cassava products.

### SHOULD CASSAVA BE REPLACED?

The advantages of the crop to the subsistence farmer, the extent of its cultivation and the significant contribution it makes to the energy intake of much of the world's population militate against an easy replacement by another staple, even if an appropriate crop could be found. Cultural resistance would be strong and one author at least believes that there would need to be severe compulsion to change to a more labour intensive crop such as rice (Hart, 1982).

### FUTURE RESEARCH

In the past the distinction between bitter and sweet varieties was felt to be blurred with certain varieties being inocuous if grown in one area and poisonous if grown in another (Lancaster et al, 1982). More recently, research has demonstrated consistently sweet varieties although these are generally limited by low yields compared to bitter varieties. Breeding experiments have suggested that a reliable high yielding sweet variety will be produced in the near future (Oben and Menz, 1981).

Further research should also be directed towards capitalising on the nutrient value of cassava leaves. Although it is acknowledged that dietary habits of people are very difficult to change, industrial means should be sought to blend cassava leaves into suitable edible forms which would entice consumer acceptance (University of Georgia, 1972).

Such developments in reducing the toxicity and improving the nutritional contribution of cassava would go a long way towards removing the dangers currently associated with cassava consumption.

### REFERENCES

ANONYMOUS (1969) New protein source found in Biafra. Food Manufacture 44, 56.

ANONYMOUS (1973) Chronic cassava toxicity. Lancet ii, 245–46.

ANONYMOUS (1979) Diabetes, cyanide and rat poison. Lancet ii, 341–2.

AYANRU, J. O. (1976) The tropical amblyopia syndrome (or tropical nutritional amblyopia) in the mid-western state of Nigeria. Afr J Med Med Sci **5**, 41–48.

BOURDOUX, P., DELANGE, F., GERARD, M., MAFUTA, M., HANSON, A and ERMANS, A. M. (1978) Evidence that cassava ingestion increased thiocyanate formation: a possible etiologic factor in endemic goiter. J Clin Endocrinol Metab **46**, 613–21.

BRAUTLECHT, C. A. (1953) Starch. Its sources, production and uses. New York, Rheinhold.

CHEOK, S. S. (1978) Acute Cassava poisoning in children in Sarawak. Tropical Doctor **8**, 99–101.

CLIFF, J., LUNDQVIST, P., MARTENSSON, J., ROSLING, H and SORBO, B (1985) Association of high cyanide and low sulphur intake in cassava-induced spastic paraparesis. Lancet ii, 1211–13.

CLUSIUS (1605) Liber Exoticum, Leyden. Cited in Bulletin of Imperial Institute 1906; **4**: 334.

COCK, J. H. (1982) Cassava: a basic energy source in the tropics. Science **218**, 755–62.

COURSEY, D. G. and HAYNES, P. H. (1970) Root crops and their potential as food in the tropics. World Crops **22**, 261–65.

HAHN, S. K. AND KEYSER, J. (1985) Cassava: a basic food of Africa. Outlook on Agriculture **14**, 95–9.

HALL, M. J. (1986) Cassava toxicity. Lancet i, 95.

HART, K. (1982) The political economy of West African agriculture. Cambridge: Cambridge University Press.

HENRY, T. A. and BOUTRON C. (1836) Cited in Bulletin of Imperial Institute **1**, 15.

INTERNATIONAL CENTRE FOR TROPICAL AGRICULTURE (1979) Annual Report, Columbia, CIAT.

JONES, W. O. (1959) Manioc in Africa. Stanford: Stanford University Press.

KELLY, F. C. and SNEDDEN, W. W. (1960) Prevalence and geographical distribution of endemic goitre. Geneva: World Health Organisation 124–26.

LANCASTER, P. A. and BROOKS, J. E. (1983) Cassava leaves as human food. Economic Botany **37**, 331–48.

LANCASTER, P. A., INGRAM, J. S., LIM, M. Y. and COURSEY, D. G. (1982) Traditional Cassava-based foods: survey of processing techniques. Economic Botany **36**, 12–45.

LUYKEN, R., De GROOT, A. P. and VAN STRATUM, P. G. C. (1961) Nutritional value of foods from New Guinea. Utrecht, Central Institute for Nutrition Food Research.

MINISTRY OF HEALTH, MOZAMBIQUE (1984) Mantakassa: an epidemic of spastic paraparesis associated with chronic cyanide intoxication in a cassava staple area. Bull WHO **62**, 477–92.

MONTGOMERY, R. D. (1980) Cyanogens. In: Liener I. E. ed. Toxic constituents of plant foodstuffs. New York: Academic Press.

NICOL, B. M. (1952) The nutrition of Nigerian peasants. Brit J Nutr, **6**, 1–5.

OBEN, D. H. and MENZ, K. M. (1981) Viewpoint: prospects for low cyanide cassava in Nigeria. Food Policy **6**, 197–200.

OKE, O. L. (1968) Cassava as food in Nigeria. Wld Rev Nutr Diet **9**, 227–50.

OKIGBO, B. N. (1980) Nutritional implications of projects giving high priority to the production of staples of low nutritive quality. Food and Nutrition Bulletin **2**, 1–10.

OSUNTOKUN, B. O. (1981) Cassava diet, chronic cyanide intoxication and neuropathy in the Nigerian Africans. Wild Rev Nutr Diet **36**, 141–73.

OYEFESO, J. A. (1976) Cassava indicated ailments—a lack of technological know-how or mere cassava consumption? The Ind J Nutr Dietet **13**, 77–83.

*(continued on page 50)*

### Abdominal symptoms in General Practice (Continued from page 34)

**REFERENCES**

### The Dangers of Cassava (Tapioca) Consumption (Continued from page 39)

PURSEGLOVE, J. W. (1974) Tropical crops: dicotyledons. London: Longman.

RICKARD, J. E. and COURSEY, D. G. (1981) Cassava storage 1. Storage of fresh cassava roots. Trop Sci **23**, 1–32.

UNIVERSITY OF GEORGIA. (1972) A literature review and research recommendations on cassava. University of Georgia.

WOOD, T. (1965) The cyanogenic glucoside content of cassava and cassava products. J Sci Food Agr **16**, 300–5.

